# Awareness of Stroke Risk Factors, Warning Signs, and Health-Seeking Behaviors Among Adults With Cardio-Metabolic Risk Attending a Primary Care Clinic in Malacca, Malaysia

**DOI:** 10.7759/cureus.86484

**Published:** 2025-06-21

**Authors:** Risq Atiqah Munirah Mohamed Mustafa, Noor Azah Abd Aziz, Mohd Fairuz Ali

**Affiliations:** 1 Department of Family Medicine, Faculty of Medicine, Universiti Kebangsaan Malaysia Medical Centre, Kuala Lumpur, MYS

**Keywords:** awareness, cardio-metabolic risk factors, health-seeking behavior, stroke, warning signs

## Abstract

Background

Early identification of stroke warning signs and risk factors may expedite intervention, thus improving the clinical outcome, which is especially important in individuals who already have risk factors. The main objective of this study is to assess awareness of stroke risk factors, warning signs, and health-seeking behavior related to stroke among adults with cardio-metabolic risk factors attending primary care.

Methods

This cross-sectional study was conducted in a primary care clinic in Malacca, Malaysia, focusing on adults with cardio-metabolic risk factors. A standardized questionnaire, piloted prior to the study, that focused on the domains of warning signs of stroke and risk factors was used, whereas the health-seeking behavior domain was adapted from the Malay version of the Attitudes and Beliefs about Cardiovascular Disease risk (ABCD-M) questionnaire. Descriptive analysis and binary logistic regression were done to analyze the factors using SPSS version 29 (IBM Corp., Armonk, NY).

Results

Out of 230 recruited patients, only 21.3% were able to identify all five stroke warning signs, with the most commonly known sign being sudden numbness or weakness on one side of the body (88.3%), comprising 203 patients, whereas only 23.9% were able to answer all 12 risk factors contributing to stroke. The mean number of warning signs known was 3.2 ± 1.4, and the mean number of risk factors known was 9.2 ± 2.8. There is a huge gap between the health-seeking behavior among this population, with the lowest mark of 8 and the highest mark of 72. The factors that contributed to health-seeking behavior related to stroke were age group (adjusted odds ratio (AOR): -0.293, 95% confidence interval (CI): 0.123-0.696), presence of heart disease (AOR: -0.237, 95% CI: 0.062-0.900), and total number of risk factors of stroke known (AOR: 1.131, 95% CI: 1.024-1.248).

Conclusion

Awareness of stroke warning signs and risk factors was still poor among the targeted population at risk of stroke. A huge gap in health-seeking behavior within this group warrants a better stroke prevention practice among this group of patients, which can be promoted by the primary healthcare provider.

## Introduction

Stroke is a non-traumatic, focused injury to the central nervous system (CNS) brought on by vascular factors. Strokes frequently result in long-term consequences, including subarachnoid hemorrhage (SAH), cerebral infarction, or intracerebral hemorrhage (ICH) ​[1,2]. Globally, around 11 million individuals live with the consequences of stroke, and in Malaysia, six new stroke cases occur every hour, comprising 52,000 cases annually [3-5]. Ischemic stroke accounts for nearly 79.4% of the cases, while hemorrhagic stroke, although less frequent (18.2%), is associated with higher mortality [2]. Individually, a stroke not only has a profound impact on the patient's quality of life but also puts a heavy strain on caretakers because of the long-term incapacity that results. Cardio-metabolic syndrome represents a constellation of metabolic abnormalities that are risk factors for cardiovascular disease, including myocardial infarction and stroke [6]. These include obesity, hypertension, dyslipidemia, and insulin resistance, which are major risk factors for stroke [7]. Recent research from the USA, Europe, Australia, and Malaysia indicates that the prevalence of these risk factors in adults is on the rise ​[3,4,8]. These conditions, once associated with older adults, are increasingly common among younger populations and contribute not only to first-time strokes but also to recurrent strokes if left unmanaged [8].

Early identification of stroke warning signs is essential for timely intervention and better outcomes. Educating the public on the warning signs and symptoms of stroke is essential, and the acronym "BEFAST" (Balance, Eyes, Face, Arm, Speech, and Time) is a useful tool to recognize early symptoms ​[9]. Rapid detection is crucial as thrombolytic therapy is time-sensitive and can significantly reduce complications [10]. Failure to recognize these signs leads to treatment delays, prolonged hospital stays, and higher morbidity and mortality rates [11]. Rising cardio-metabolic risk factors further emphasize the need to educate individuals about both modifiable and non-modifiable stroke risk factors to improve early detection and prevention.

Health-seeking behavior for stroke is strongly influenced by an individual's awareness and perception of stroke symptoms [11,12]. Studies in Malaysia have shown low levels of public awareness regarding stroke warning signs and appropriate responses. For example, a 2014 survey in Selangor, Malaysia, by Deen et al. revealed that none of the participants knew about acute stroke treatment availability, and only 35% could identify all five warning signs [12]. Although a 2019 nationwide urban study among 4,096 respondents across 42 centers showed improved awareness, 74.3% were able to recognize all five stroke symptoms, especially among the elderly with comorbidities and previous stroke experience; a small proportion (4%) still could not recognize any stroke symptoms [11]. These findings were biased as respondents with a prior history of stroke affected the results, and the focus on the level of awareness among those at risk of stroke could not be elicited from the study. Despite some local studies, limited research has focused specifically on individuals with cardio-metabolic risk factors, the group most vulnerable to stroke.

Health behavior theories such as the Health Belief Model (HBM) and Transtheoretical Model (TTM) suggest that individuals are more likely to adopt preventive behaviors when they perceive themselves at risk and believe in the benefits of action [13]. Lack of knowledge about stroke symptoms and its awareness among the Asian population has led to a delay in seeking medical attention, hence increasing morbidity and mortality [14]. A local study on assessing the level of awareness using the Attitudes and Beliefs about Cardiovascular Disease (ABCD) risk questionnaire has shown that awareness of stroke risk remains only moderately satisfactory among the general Malay population, with a mean score of 49.26 from a total mark of 80 [15]. Similarly, research from China showed many individuals with high cardiovascular risk underestimated their likelihood of experiencing a cardiovascular event [16]. These findings emphasize the need to assess stroke awareness and health-seeking behaviors specifically in people with cardio-metabolic risk factors.

Rising prevalence of stroke warrants a focus on the primary prevention for those with cardio-metabolic characteristics and secondary prevention for those who are unaware of the risk of developing another stroke. Thus, it is important to assess those with cardio-metabolic risk factors of their level of awareness of stroke risk factors and warning signs, and their perceived health-seeking behavior for developing stroke. Most local studies focused on the overall population and rural areas, but none on the targeted group of people with cardio-metabolic risk factors. The gaps identified from this study will help primary care doctors and administrators to improve primary prevention of stroke in Malaysia in line with the Health White Paper for Malaysia Pillar 2, which advocates for strengthened health promotion and disease prevention strategies [17].

## Materials and methods

Study design, population, and sample size

This cross-sectional study was conducted among adults with cardio-metabolic risk factors attending a primary care clinic in Malacca, Malaysia, between June and December 2024. Participants were recruited using convenience sampling. Inclusion criteria include those aged 18 years and above who met the study criteria (having at least one cardio-metabolic risk factor, i.e., hypertension, diabetes mellitus, dyslipidemia, obesity or overweight, smoking, and alcohol consumption) or a known diagnosis of heart disease and those who consented to the study. Exclusion criteria include individuals with a history of stroke or transient ischemic attack and those presenting with acute presentation of warning signs of stroke or have any condition that makes them unable to participate, e.g., visual impairment, cognitive impairment, or physical impairment.

The sample size was calculated using a confidence level of 95% with a margin of error of 5%, based on a simple mean formula from a previous study ​[15]​. Considering a 20% dropout rate, the recommended total sample size was 215. We recruited a total of 230 participants to this study after excluding almost 20 incomplete questionnaires.

Research tools

A structured, self-administered questionnaire was used in this study. It is a three-part questionnaire assessing the following: part 1, awareness of stroke warning signs; part 2, stroke risk factors; and part 3, health-seeking behavior. Parts 1 and 2 were adapted with permission from the study by Ahmed et al. (2019) [9], with internal consistency of Cronbach's alpha of 0.79 (English) and 0.83 (Malay).

The scores for all five warning sign items were summed to obtain the overall score for each part. Total warning signs known score ranged between 0 and 5, and was also categorized into "known all five warning signs of stroke" or "not known all five warning signs of stroke." Higher scores indicate better awareness of the warning signs of stroke. No negative marking was given for the wrong choice of warning signs of stroke. The total number of risk factors known score ranges between 0 and 12 and was categorized into "known all 12 risk factors of stroke" or "not known all 12 risk factors of stroke." Higher scores indicate better awareness of the risk factors of stroke. No negative marking was given for the wrong choice of risk factors of stroke ​[9].

Part 3 was adapted, with permission, from the Attitudes and Beliefs about Cardiovascular Disease (ABCD-M) risk questionnaire, which comprises 26 questions. It has four domains, with a score of 0-80, and has been translated into the Malay language by Mat Said et al. ​[15]. The internal consistency and composite reliability of the domains showed good results ranging from 0.643 to 0.885, hence making it a valid and reliable tool to assess health-seeking behavior for stroke and CVD risk in the Malaysian population. A pilot study was done prior to actual data collection to determine the internal consistency of the questionnaire after combining the three parts of the questionnaire. The overall Cronbach's alpha of the questionnaire was 0.71 after removing domain 1 of the ABCD-M original questionnaire in view of repetitive questions. The term "heart attack" was also removed to align with the focus of the study on awareness of stroke.

There are three domains in the health-seeking behavior questionnaire. Domain 1 is about perceived risk of stroke (total: eight items) using a Likert scale ranging from 0 to 4 for each item (strongly disagree to strongly agree), giving total marks of 0-32 for domain 1; a higher sum score shows a higher perception of risk of having a stroke. One question is reverse-coded, which is question 7. Higher scores indicate a better perceived risk of stroke. Domain 2 is about perceived benefits (total: four items) using a Likert scale ranging from 0 to 4 for each item (strongly disagree to strongly agree), giving total marks of 0-16 for domain 2; a higher sum score shows a higher perceived benefit of diet and exercise. Domain 3 is about intention to change (total: six items) using a Likert scale ranging from 0 to 4 for each item (strongly disagree to strongly agree), giving total marks of 0-24 for domain 3; a higher sum score shows a higher perceived readiness for changes regarding exercise behavior and healthy dietary behavior. Two questions are reverse-coded, which are questions 15 and 18. The scores were summed for each domain. The total marks from each domain will be calculated and combined, ranging from 0 to 72. The marks were grouped into poor health-seeking behavior (0-24), moderate health-seeking behavior (25-54), and good health-seeking behavior (55-72). Scoring was made after discussion with experts in the field, as the data were not normally distributed.

Data collection

Data collection was carried out between June and December 2024. Participants were patients attending follow-ups at the quit smoking clinic, non-communicable disease clinic, pharmacy visits, and outpatient visits. Potential participants were approached at the waiting area and were briefed on the study before giving their consent. Written informed consent was obtained, instruction was given on how to complete the questionnaire, and the participants were given time to answer the questionnaire. Researcher and clinic staff who agreed to be enumerators were available to guide patients in answering the questionnaire.

Statistical analysis

Data were analyzed using SPSS version 29 (IBM Corp., Armonk, NY). Descriptive analysis of the variables was conducted using frequency and percentage for categorical data, and mean and standard deviation (SD) were used for continuous data. Factors associated with health-seeking behavior were analyzed using binary logistic regression after the behavior was dichotomized into two categories: poor and moderate health-seeking behavior, and good health-seeking behavior, based on statistical consultation. All independent variables were included in the study. Variables with a p-value < 0.25 were considered significant and were included in the final analysis. A p-value of <0.05 is considered statistically significant.

Ethical approval

This study was approved by the Universiti Kebangsaan Malaysia Research Ethics Committee on March 13, 2024 (JEP-2024-123) and by the Medical Research and Ethics Committee (MREC), Ministry of Health Malaysia (MOH) (NMRR ID-24-00581-MNP). Written consent was obtained from the participants who agreed to participate.

## Results

Sociodemographic and clinical characteristics of the study participants

A total of 230 completed questionnaires were included in the study. Table 1 shows the sociodemographic data and clinical profile of the participants included. The mean age of the participants in this study was 53.63 ± 13.66 years, ranging from 23 to 80 years old. The mean body mass index (BMI) was 29.56 ± 6.16 kg/m^2^, ranging from 17.2 to 47.4 kg/m^2^, with a mean weight of 77.27 ± 19.08 kg, ranging from 43.0 to 145.0 kg. For the total risk factor, the participants have shown a mean of 3.47 ± 1.35. In terms of total warning signs known by the participants, those with no formal education have the lowest mean value of 1.60 ± 1.517; the other groups have almost similar mean numbers of stroke warning signs known.

**Table 1:** Sociodemographic and clinical characteristic of the study participants (N = 230) HSB: health-seeking behavior, BMI: body mass index

Characteristic	HSB			Total (number (%))
	Poor HSB (number (%))	Moderate HSB (number (%))	High HSB (number (%))	
Sociodemographic profile				
Age group	0 (0.0)	11 (4.8)	25 (10.9)	36 (15.7)
Young adult (18-39 years)				
Middle-aged adult (40-59 years)	1 (0.4)	46 (20.0)	61 (26.5)	108 (47.0)
Older adult (60-80 years)	2 (0.9)	47 (20.4)	37 (16.1)	86 (37.4)
Gender	2 (0.9)	47 (20.4)	57 (24.8)	106 (46.1)
Male				
Female	1 (0.4)	57 (24.8)	66 (28.7)	124 (53.9)
Ethnicity	2 (0.9)	72 (31.3)	92 (40.0)	166 (72.2)
Malay				
Chinese	1 (0.4)	19 (8.3)	21 (9.1)	41 (17.8)
Indian	0 (0.0)	10 (4.3)	10 (4.3)	20 (8.7)
Others	0 (0.0)	3 (1.3)	0 (0.0)	3 (1.3)
Marital status	0 (0.0)	10 (4.3)	14 (6.1)	24 (10.4)
Single				
Married	3 (1.3)	88 (38.3)	102 (44.3)	193 (83.9)
Divorced/widowed	0 (0.0)	6 (2.6)	7 (3.0)	13 (5.7)
Education level	0 (0.0)	2 (0.9)	3 (1.3)	5 (2.2)
No formal education				
Primary education	0 (0.0)	8 (3.5)	4 (1.7)	12 (5.2)
Secondary education	1 (0.4)	61 (26.5)	64 (27.8)	126 (54.8)
Tertiary education	2 (0.9)	33 (14.3)	52 (22.6)	87 (37.8)
Employment status	0 (0.0)	21 (9.1)	16 (7.0)	37 (16.1)
Housewife				
Self-employed	0 (0.0)	14 (6.1)	13 (5.7)	27 (11.7)
Government/private	1 (0.4)	37 (16.1)	60 (26.1)	98 (42.6)
Retired	2 (0.9)	25 (10.9)	30 (13.0)	57 (24.8)
Unemployed	0 (0.0)	7 (3.0)	4 (1.7)	11 (4.8)
Monthly income	0 (0.0)	57 (24.8)	45 (19.6)	102 (44.3)
< RM 2,500				
B40: RM 2,500-RM 4,849	1 (0.4)	35 (15.2)	55 (23.9)	91 (39.6)
M40: RM 4,850-RM 10,959	2 (0.9)	12 (5.2)	22 (9.6)	36 (15.7)
T20: >RM 10,960	0 (0.0)	0 (0.0)	1 (0.4)	1 (0.4)
Clinical profile				
Diabetes mellitus	1 (0.4)	51 (22.2)	49 (21.3)	101 (43.9)
Yes				
No	2 (0.9)	53 (23.0)	74 (32.2)	129 (56.1)
Hypertension	1 (0.4)	55 (23.9)	70 (30.4)	126 (54.8)
Yes				
No	2 (0.9)	49 (21.3)	53 (23.0)	104 (45.2)
High cholesterol	1 (0.4)	56 (24.3)	80 (34.8)	137 (59.6)
Yes				
No	2 (0.9)	48 (20.9)	43 (18.7)	93 (40.4)
Heart disease	0 (0.0)	3 (1.3)	12 (5.2)	15 (6.5)
Yes				
No	3 (1.3)	101 (43.9)	111 (48.3)	215 (93.5)
Smoker/vape	0 (0.0)	19 (8.3)	20 (8.7)	39 (17.0)
Yes				
No	3 (1.3)	85 (37.0)	103 (44.8)	191 (83.0)
BMI classification	0 (0.0)	1 (0.4)	2 (0.9)	3 (1.3)
Underweight: <18.5 kg/m^2^				
Normal: 18.5-22.9 kg/m^2^	0 (0.0)	11 (4.8)	17 (17.4)	28 (12.2)
Pre-obese: 23-27.4 kg/m^2^	1 (0.4)	41 (17.8)	25 (10.9)	67 (19.1)
Obese: >27.5 kg/m^2^	2 (0.9)	51 (22.2)	79 (34.3)	132 (57.4)
Physical activity	0 (0.0)	28 (12.2)	24 (10.4)	52 (22.6)
Active				
Inactive	3 (1.3)	76 (33.0)	99 (43.0)	178 (77.4)
Alcohol intake	0 (0.0)	0 (0.0)	1 (0.4)	1 (0.4)
Excessive alcohol intake				
Normal alcohol intake	0 (0.0)	7 (3.0)	4 (1.7)	11 (4.8)
Non-alcoholic	3 (1.3)	97 (42.2)	118 (51.3)	218 (94.8)
Total risk factor	0 (0.0)	6 (2.6)	7 (3.0)	13 (5.7)
1 risk factor only				
2-4 risk factors	3 (1.3)	74 (32.2)	83 (36.1)	160 (69.6)
5-8 risk factors	0 (0.0)	24 (10.4)	33 (14.3)	57 (24.8)

Awareness of stroke warning signs and risk factors

**Figure 1:**
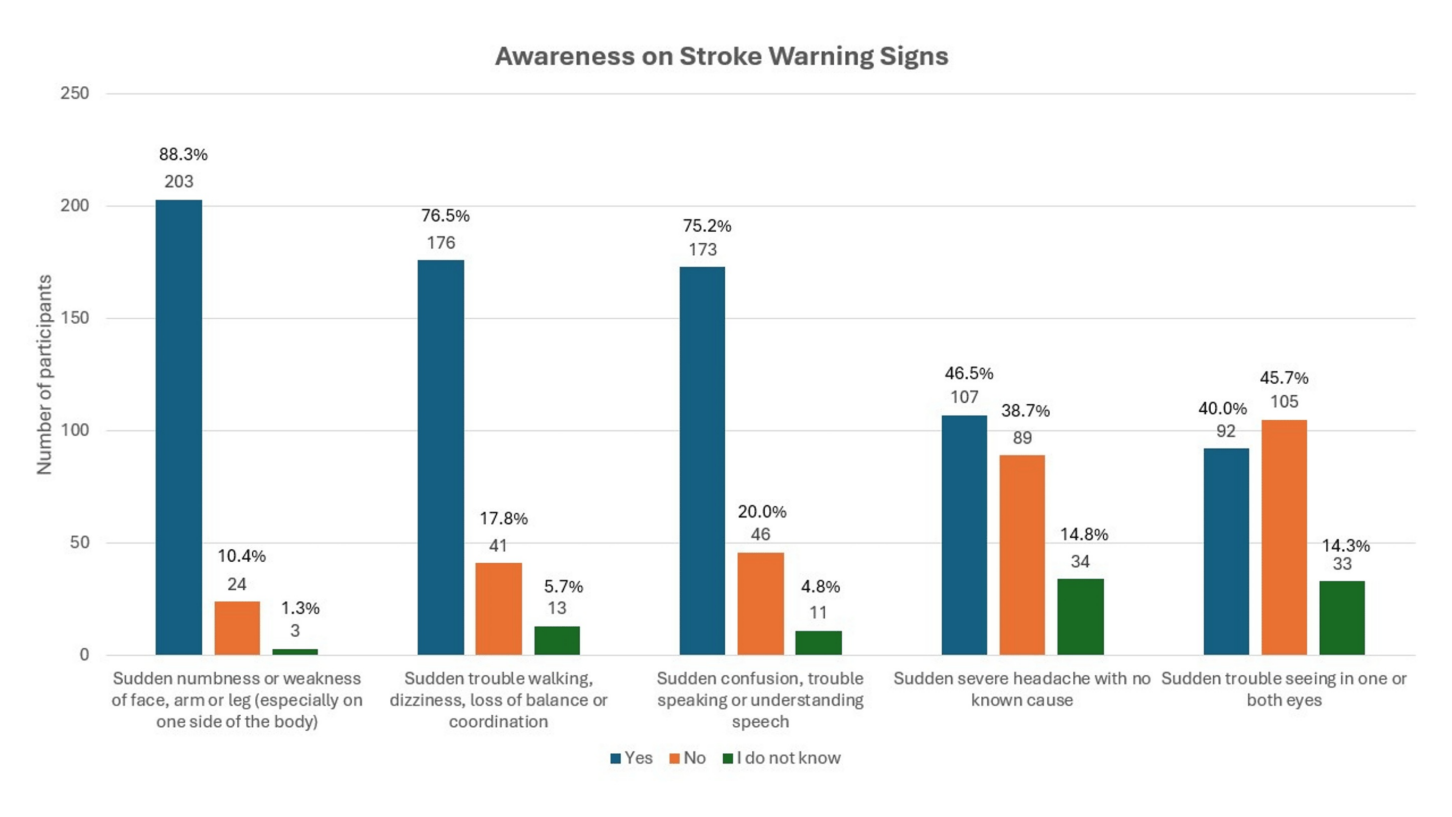
Bar chart on awareness on stroke warning signs known by the study participants (N = 230)

**Figure 2:**
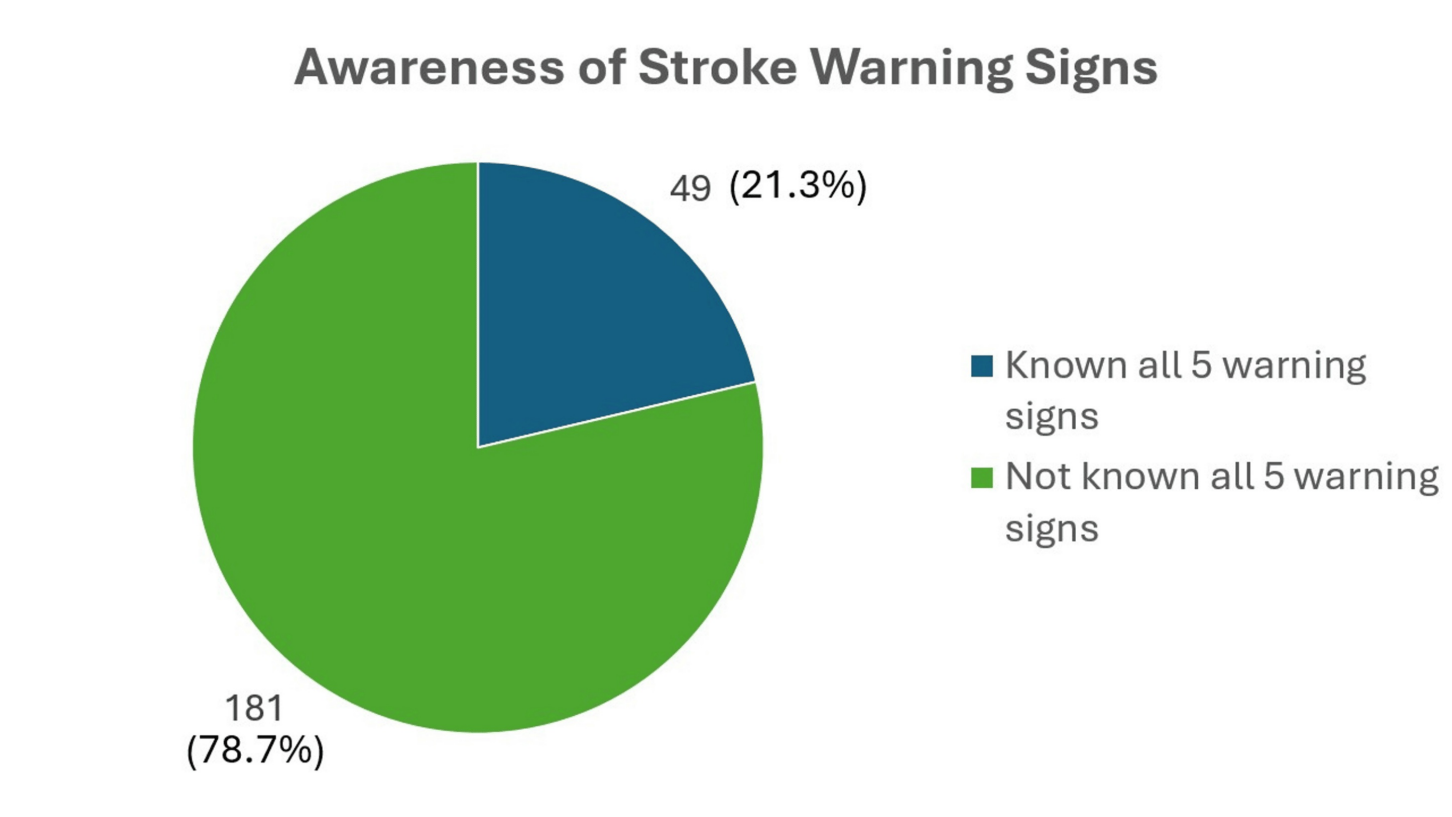
Pie chart on awareness of stroke warning signs (N = 230)

Figure 1 and Figure 2 report on the warning signs of stroke, in which the study shows that only 49 (21.3%) participants knew all five warning signs of stroke, with sudden weakness being the most recognizable (203 (88.3%)), followed by sudden severe headache with no known cause (107 (46.5%)), and with sudden trouble seeing in one or both eyes (92 (40.0%)) being the least recognizable. The mean number of warning signs known by the participants was 3.28 ± 1.44. Surprisingly, 21 (9.1%) participants were unable to recognize any stroke warning signs.

**Figure 3:**
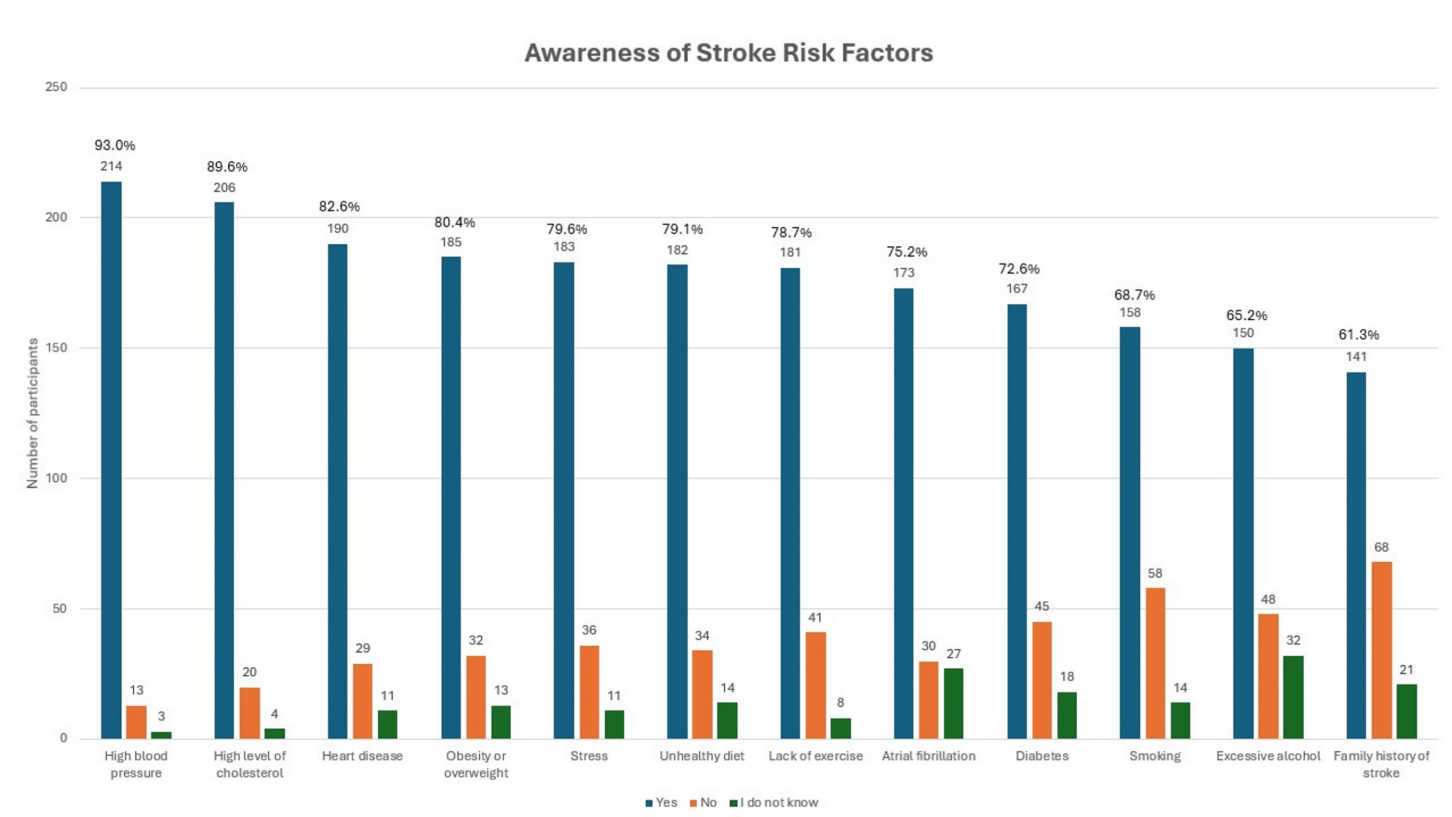
Bar chart on awareness of stroke risk factors known by the study participants (N = 230)

**Figure 4:**
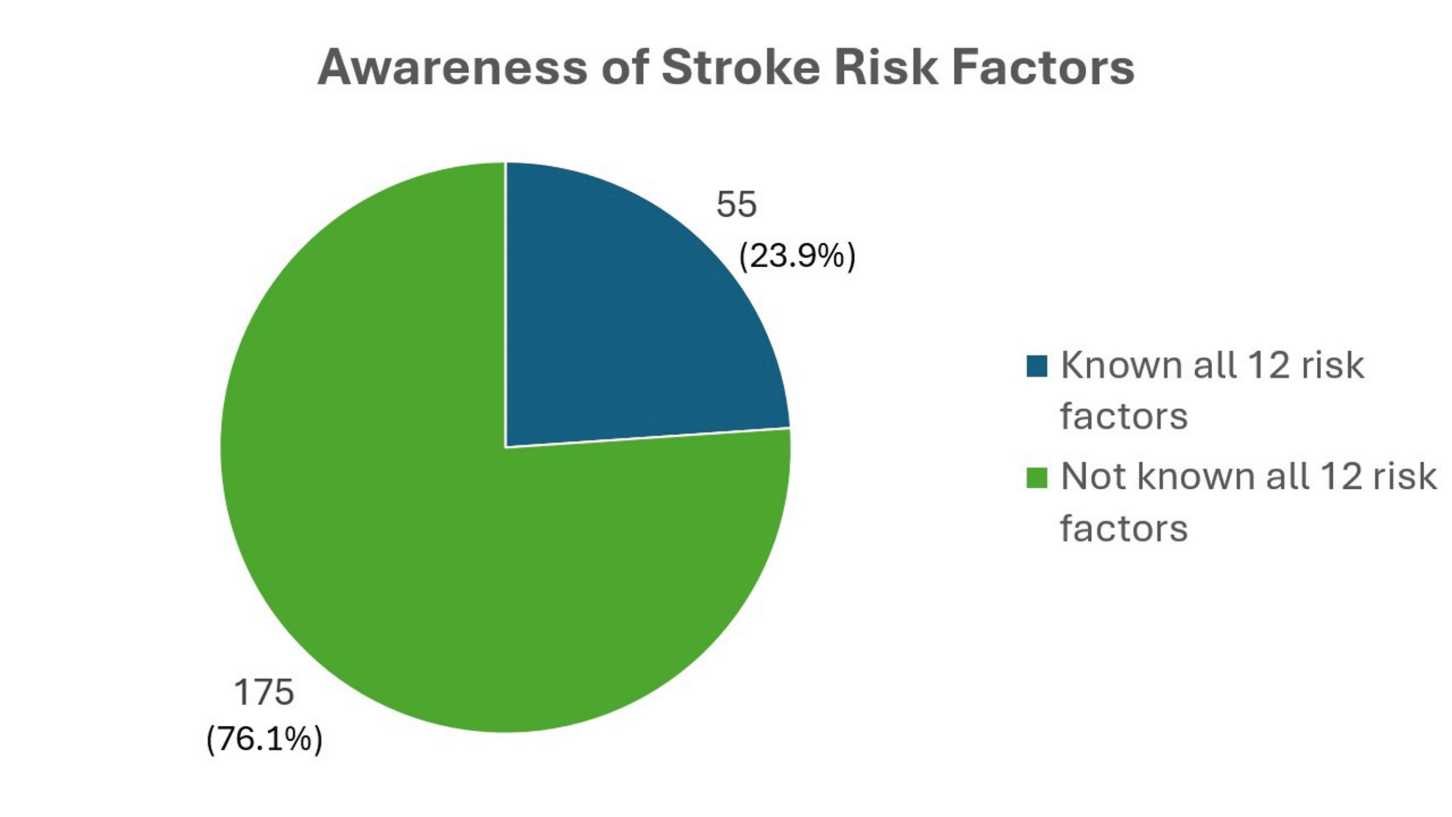
Pie chart on awareness of stroke risk factors (N = 230)

Figure 3 and Figure 4 report on the risk factors of stroke known by the participants. The majority of the participants (175 (76.1%)) were not aware of all 12 risk factors of stroke, and only 55 (23.9%) were able to recognize all 12 risk factors of stroke. The mean number of stroke risk factors known by the participants was 9.26 ± 2.87. However, only one (0.4%) participant was unable to recognize at least one risk factor of stroke. The least recognizable risk factors are smoking (158 (68.7%)), excessive alcohol intake (150 (65.2%)), and family history of stroke (141 (61.3%)). The most known risk factor of stroke known by the participants was high blood pressure (214 (93.0%)), followed by high levels of cholesterol (206 (89.6%)), and the least known was a family history of stroke. Despite being one of the biggest contributors to developing cardiovascular disease, including stroke, diabetes was recognized as a stroke risk factor by only 167 (72.6%) participants.

Health-seeking behavior for stroke

**Figure 5:**
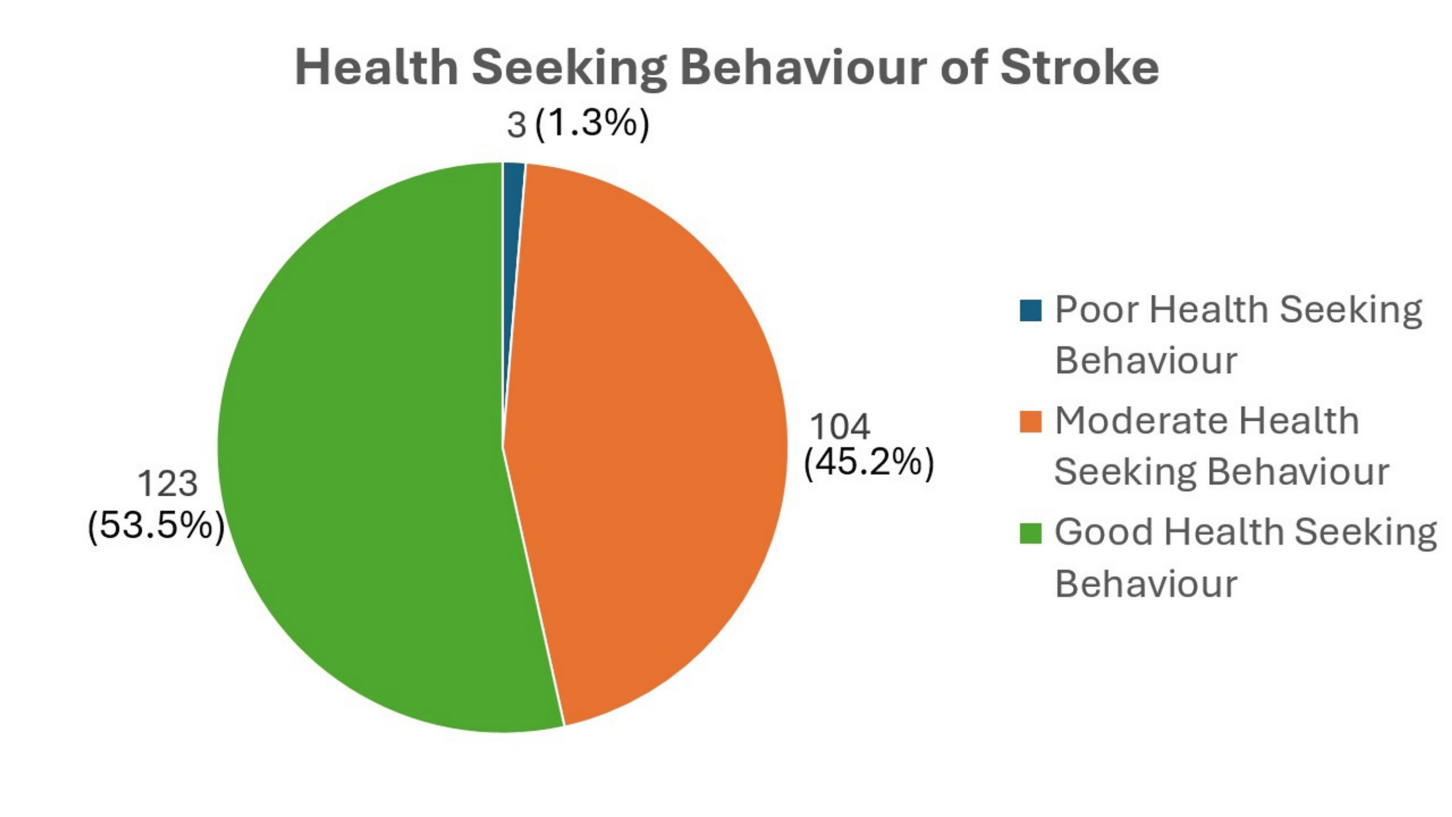
Pie chart on health-seeking behavior for stroke (N = 230)

Table 2 and Figure 5 report on the domain health-seeking behavior for stroke. Between 0 and 8 participants selected "not applicable" as their response for perceived risk factors of stroke and perceived benefits. This is alarming as they are considered low health-conscious participants. On the other hand, the total mean marks of all domains of health-seeking behavior were 54.84 ± 9.94; however, the lowest marks scored by the participants showed a huge gap, ranging from 8 to 72, with a higher score indicating better health-seeking behavior.

**Table 2:** Domain for health-seeking behavior for stroke HSB: health-seeking behavior, SD: standard deviation

Items	Item scoring					Total marks HSB (mean ± SD) (range)
	Not applicable (number (%))	Strongly disagree (number (%))	Disagree (number (%))	Agree (number (%))	Strongly agree (number (%))	
Domain 1: Perceived risk factors of stroke						
1. I feel that I will suffer from a stroke sometime during my life.	8 (3.5)	35 (15.2)	92 (40.0)	7 (31.3)	23 (10.0)	20.07 ± 6.76 (0-32)
2. It is likely that I will suffer from a stroke in the future.	8 (3.5)	36 (15.7)	85 (37.0)	78 (33.9)	23 (10.0)	
3. It is likely that I will have a stroke sometime during my life.	8 (3.5)	37 (16.1)	83 (36.1)	80 (34.8)	22 (9.6)	
4. There is a good chance I will experience a stroke in the next 10 years.	7 (3.0)	39 (17.0)	89 (38.7)	73 (31.7)	22 (9.6)	
5. My chances of suffering from a stroke in the next 10 years are great.	7 (3.0)	38 (16.5)	88 (38.3)	74 (32.2)	23 (10.0)	
6. It is likely that I will have a stroke because of my past and/or present behaviors.	8 (3.5)	24 (10.4)	86 (37.4)	87 (37.8)	25 (10.9)	
7. I am not worried that I might have a stroke (reverse-coded).	8 (3.5)	110 (47.8)	76 (33.0)	27 (11.7)	9 (3.9)	
8. I am concerned about the likelihood of having a stroke in the near future.	12 (5.2)	20 (8.7)	29 (12.6)	79 (34.3)	90 (39.1)	
Domain 2: Perceived benefits						
9. When I exercise for at least 2 ½ hours a week, I am doing something good for the health of my heart.	3 (1.3)	0 (0.0)	4 (1.7)	49 (21.3)	174 (75.7)	14.30 ± 2.17 (0-16)
10. I am confident that I can maintain a healthy weight by exercising at least 2 ½ hours a week within the next two months.	1 (0.4)	1 (0.4)	29 (12.6)	89 (38.7)	110 (47.8)	
11. When I eat at least five portions of fruit and vegetables a day, I am doing something good for the health of my heart.	2 (0.9)	1 (0.4)	8 (3.5)	54 (23.5)	165 (71.7)	
12. Increasing my exercise to at least 2 ½ hours a week will decrease my chances of having a stroke.	2 (0.9)	2 (0.9)	7 (3.0)	66 (28.7)	153 (66.5)	
Domain 3: Intention to change						
13. I am thinking about exercising at least 2 ½ hours a week.	0 (0.0)	1 (0.4)	24 (10.4)	97 (42.2)	108 (47.0)	20.45 ± 3.39 (0-24)
14. I intend or want to exercise at least 2 ½ hours a week.	0 (0.0)	0 (0.0)	27 (11.7)	95 (41.3)	108 (47.0)	
15. I am not thinking about exercising for 2 ½ hours a week (reverse-coded).	4 (1.7)	127 (55.2)	66 (28.7)	31 (13.5)	2 (0.9)	
16. I am confident that I can eat at least five portions of fruits and vegetables per day within the next two months.	1 (0.4)	2 (0.9)	19 (8.3)	77 (33.5)	131 (57.0)	
17. I am thinking about eating at least five portions of fruits and vegetables a day.	1 (0.4)	2 (0.9)	16 (7.0)	82 (35.7)	129 (56.1)	
18. I am not thinking about eating at least five portions of fruits and vegetables a day (reverse-coded).	4 (1.7)	146 (63.5)	57 (24.8)	21 (9.1)	2 (0.9)	

Spearman's rho correlation analysis was done to determine the relationship between the mean total number of stroke warning signs known and the mean score of total health-seeking behavior for stroke, which shows a weak positive correlation and was statistically significant (rs = 0.169, p = 0.010). Spearman correlation was also done to determine the relationship between the mean total number of risk factors known and the mean score of total health-seeking behavior for stroke, which shows a weak positive correlation and was statistically significant (rs = 0.177, p = 0.007).

Factors associated with health-seeking behavior for stroke

A binary logistic regression analysis was conducted after health-seeking behavior was dichotomized into two categories (poor and moderate health-seeking behavior, and good health-seeking behavior) to investigate the effect of age group, diabetes mellitus, heart disease, and total risk factor on the likelihood of one implementing good health-seeking behavior. The model was statistically significant (χ^2^ (5) = 22.211, p < 0.001), explaining between 9.2% (Cox and Snell R-squared) and 12.3% (Nagelkerke R-squared) of the variance in sociodemographic factor/clinical background and correctly classifying 64.8% of cases. The Hosmer-Lemeshow test suggested a good fit to the data (χ^2^ (8) = 6.821, p = 0.556). This model reveals that the odds of demonstrating good health-seeking behavior are reduced significantly by 0.3 times in older age groups compared to young adults (p < 0.05). The odds of demonstrating good health-seeking behavior in those without heart disease are reduced by 0.3 times compared to those with one (p = 0.034). With every unit change of total risk factor known, it costs a significant increment of good health-seeking behavior by one unit.

**Table 3:** Summary of binary logistic regression analysis of health-seeking behavior based on independent variables *Statistically significant OR: odds ratio, CI: confidence interval

Variables	Categories	Adjusted OR	95% CI	p-value
Age group	Young	Reference	-	0.010*
	Middle	-0.580	0.251-1.338	0.202
	Older	-0.293	0.123-0.696	0.005*
Diabetes mellitus	Diabetic	Reference	-	-
	Non-diabetic	1.381	0.788-2.420	0.259
Heart disease	Yes	Reference	-	-
	No	-0.237	0.062-0.900	0.034*
Total number of stroke risk factor known	-	1.131	1.024-1.248	0.015*

## Discussion

Awareness of stroke warning signs

The rising prevalence of non-communicable disease warrants better stroke prevention, especially by primary care physicians, to help reduce the stroke burden in Malaysia. The Health White Paper for Malaysia, published in 2023, aims to improve primary prevention of stroke in Malaysia, in line with Pillar 2, which aims for the advancement of health promotion and disease prevention​ [17].

Our current study on adults with cardio-metabolic risk factors shows a moderate level of awareness of stroke warning signs, with a mean of 3.28 ± 1.44. However, what is more concerning is that only 21.3% (around 49 participants from the study) knew all five warning signs of stroke. This is considered poor, as the study was done among a targeted population that should show a high level of awareness of stroke warning signs, as they are vulnerable to the disease. Compared to a previous study conducted on the rural population of Selangor, the level of awareness was better, in which around 36.44% were able to identify all five warning signs of stroke ​[18]. A local study conducted among the general population in an urban setting using community-based surveys also shows a relatively poor level of awareness, where only 35% of the study respondents were able to identify stroke symptoms correctly ​[12]. The multicenter study conducted by Ching et al. on a general population of Malaysia, which consists of 42 centers, shows better results, in which 74.3% of the study respondents successfully identified all five symptoms of stroke, despite having 4% who were unable to recognize even one stroke symptom ​[11]. Compared to the current study, the percentage of participants in the targeted population who could not identify at least one stroke symptom was significantly higher, with 9.1% (21 participants) being unable to recognize even one stroke symptom. Nevertheless, the result was much better than the findings from a local community-based survey, which shows that almost a quarter of the respondents were unable to recognize even a single warning sign (27%) ​[12]. A study conducted in Nepal among high school students shows that only 5.2% of the respondents knew all stroke warning signs; nevertheless, only 2.5% of the respondents were unable to recognize any of the stroke symptoms ​[19]. In comparison, in a study conducted in Islamabad among a targeted population of hypertensive patients, the mean number of warning signs identified was only 1.5, and none of the participants were able to identify stroke warning signs, similar to a study conducted in India, where none of the participants knew more than one warning sign of stroke ​[20,21]. Although the current study shows better awareness of stroke warning signs among the targeted population as compared to studies from other countries, the fact that the percentage is still low warrants an aggressive preventive strategy to improve stroke prevention.

The study conducted by Ching et al. among the general population in a multicenter setting in Malaysia found that 90.3% of the participants identified weakness of one side of the body as a stroke warning sign [11], almost similar to the current study's finding of 88.3%. The study conducted in Islamabad among hypertensive patients also recognized sudden onset numbness of limbs as the most common warning sign of stroke, with 66.9% of participants able to identify it as one of the stroke warning signs; this is comparable to a study in Thailand, with 92% of participants able to recognize sudden unilateral numbness and weakness as stroke warning signs ​[20,22]. The current study found that the least known stroke warning sign was sudden trouble seeing in one or both eyes, which was known by only 40% of the study participants. This rate is lower in comparison to the study conducted among the rural population in Selangor (62.4%) and the study in Thailand among diabetic and hypertensive patients, where 59.6% recognized it as one warning sign of a stroke ​[18,22]. The study done in Indonesia among hypertensive, diabetic, and heart failure patients also showed that 65.7% were unaware that visual disturbances in one or both eyes are warning signs of a stroke ​[23]. This is a similar finding to a study on stroke awareness conducted in Turkey among hypertensive patients, where only 32% recognized loss of vision as one of stroke warning signs ​[24]. Our study showed that those with low socioeconomic status, with a monthly income of <RM 2,500, are less likely to recognize all stroke warning signs, where only 22 out of 102 (21.5%) participants are able to recognize all stroke warning signs. Only six out of 36 (16.6%) participants in the younger age group were able to recognize all five stroke warning signs. This is in contrast with studies done in Ethiopia, Iraq, and Nigeria, where younger people better understand stroke symptoms ​[25-27].

Awareness of stroke risk factors

The most common risk factor known to cause stroke among the current participants was high blood pressure (93%), which is consistent with other studies conducted in Indonesia, Nigeria, and Islamabad ​[20,23,28]. The study done in Indonesia on patients with hypertension, diabetes, and heart failure also shows similar findings as the current study, whereby diabetes mellitus (42.4%), smoking (40.4%), and alcohol consumption (38.4%) are the most common risk factors that participants are unaware about ​[23]. A study conducted in Portugal also revealed similar results, as only 15.4% of the participants were able to spontaneously recall diabetes as one of stroke risk factors ​[29]. In the current study, the least commonly known risk factors of stroke were diabetes (72.6%), smoking (68.7%), excessive alcohol intake (65.2%), and family history of stroke (61.3%). This enlightens us to improve our preventive strategies to target more of this targeted group, especially diabetic patients, as most respondents do not recognize diabetes as a of stroke, to emphasize that it is one of the risk factors for developing stroke. A campaign through social media and mass media would be helpful in giving information about important risk factors contributing to stroke. This is also supported by the fact that diabetes is expected to affect seven million Malaysian adults aged 18 years and above by the year 2025 ​[18]. Although there was only weak positive association between the total number of risk factors known with health-seeking behavior from Spearman rho's correlation study, the total number of risk factors known is one of the predictors of having good health-seeking behavior, as every unit change of the total risk factor known results in a significant increment of good health-seeking behavior by one unit.

Health-seeking behavior for stroke

Health-seeking behavior for stroke was adapted by the Health Belief Model, a holistic approach to disease prevention methods ​[13]. The three domains tested in the current study were perceived risk factors of stroke, perceived benefits, and intention to change. Surprisingly, in our study, almost half of the participants had negative health-seeking behavior as they did not perceive that they were at risk of developing a stroke, although they belonged to the targeted group. The mean score of the perceived risk factors of stroke is quite low, with only 20 out of 32 marks in the domain (62.5%). The younger age group demonstrated better health-seeking behavior, with a mean of 57.38 ± 9.741; this was statistically significant, as the older age group showed a significant decrease of 0.3 in comparison. The presence of heart disease was also statistically significantly associated with better health-seeking behavior, as the mean of health-seeking behavior was also higher in heart disease patients (60.47 ± 6.760), in comparison to those without heart disease (54.45 ± 10.018). In contrast, the local study on stroke awareness conducted on a general population found a score range of 6-77, as they used a four-domain questionnaire; this result is similar to the current study. The domain of perceived risk of heart attack or stroke had the lowest mean score, at 14.97 ± 6.01, comprising 46.78% of the total marks​ [15]. As previously explained, the domain of knowledge of stroke was amended in view of repetitive questions. Nevertheless, this shows that many of the respondents are not aware that they are at risk of developing a stroke. This assessment of stroke awareness is essential to provide a clearer picture of how participants at risk of developing stroke perceive their condition. This study gives us some insight to ensure that people at risk engage in good health-seeking behavior, which helps motivate them to seek treatment earlier and to be more engaged in taking care of their own health.

Strengths and limitations of the study 

Our study highlights the importance of assessing awareness of stroke warning signs and risk factors, alongside the health-seeking behavior for stroke among adults with cardio-metabolic risk factors, a population particularly vulnerable to stroke. A key strength of this research lies in its targeted approach, focusing on a high-risk group that is often underrepresented in stroke awareness studies within the Malaysian context. Furthermore, local data on stroke-related health-seeking behavior remains limited, adding value to the findings of this study.

However, several limitations should be acknowledged. This study was conducted in a single public primary care clinic in Malacca using convenience sampling, which may introduce selection bias and limit the generalizability of the findings to the wider Malaysian population. The sociodemographic profile of the respondents, such as the predominance of middle-aged adults with existing comorbidities, may not fully represent other subpopulations, including younger adults. Future research involving a multicenter, nationally representative sample is recommended to gain deeper insights into stroke awareness trends among the targeted population in Malaysia.

Study implication

The current study showed that there is a huge gap in managing patients with comorbid conditions who are at risk of developing a stroke. More intervention and educational materials should be emphasized among patients at risk. Using pamphlets and targeted outreach programs among those at risk may increase the awareness of stroke, especially in primary care settings. This study might be considered a foundation for future research in which we can assess awareness pre- and post-interventional programs to assess the impact of education programs on stroke awareness among targeted populations.

## Conclusions

Awareness of stroke warning signs and risk factors remains poor, particularly among the high-risk population. While most participants recognize that sudden numbness or weakness on one side of the body is a key warning sign, fewer than half are aware that sudden trouble seeing in one or both eyes or a sudden severe headache with no known cause can also indicate a stroke. Healthcare practitioners should place greater emphasis on educating at-risk individuals, particularly on the early detection of stroke symptoms, to improve outcomes.

The significant gap in health-seeking behavior within this group highlights the need for improved stroke prevention practices, which can be effectively promoted through primary healthcare providers. Additionally, the poor perceived risk of stroke among these patients is concerning, as many do not recognize their vulnerability. This presents a crucial opportunity to implement more targeted stroke awareness campaigns in primary care clinics across Malaysia, especially in targeted populations.

## Appendices

Questionnaire

Section A: Sociodemographic Characteristics

Bahagian A: Ciri-ciri socio-demografi

Please answer the following questions. Please ✔ on the appropriate box.

Sila jawab soalan berikut. Sila tanda ✔ pada kotak yang berkenaan

Age (in years): ____________________

Umur (dalam tahun): ____________________

Table 4 presents the sociodemographic profile questionnaire.

**Table 4:** Sociodemographic profile Credits: Risq Atiqah Munirah binti Mohamed Mustafa

Gender/Jantina	
Male/Lelaki	
Female/Perempuan	
Ethnicity/Bangsa	
Malay/Melayu	
Chinese/Cina	
Indian/India	
Others/Lain-lain	
Marital status/Status perkahwinan	
Single/Bujang	
Married/Berkahwin	
Divorced/Widowed (Bercerai/Duda/Balu)	
Education level/Tahap pendidikan	
No formal education/Tiada pendidikan formal	
Primary education/Pendidikan rendah	
Secondary education/Pendidikan menengah	
Tertiary education/Pendidikan tinggi	
Employment status/Status pekerjaan	
Housewife/Surirumah	
Self-employed/Bekerja sendiri	
Government/Private (Pekerja kerajaan/swasta)	
Retired/Pesara	
Unemployed/Tidak bekerja	
Monthly income/Pendapatan bulanan	
< RM 2,500	
B40: RM 25,00-RM 4,849	
M40: RM 4,950-RM 10,959	
T20: >RM 10,960	

Section B: Clinical Profile (Cardio-Metabolic Risk Factors)

Bahagian B: Profil Klinikal (Faktor risiko kardio-metabolik)

Height (in cm)/Tinggi (dalam cm): ________________________

Weight (in kg)/Berat (dalam kg): ________________________

BMI (kg/m^2^): ________________________

Please answer the following questions. Please ✔ on the appropriate box.

Sila jawab soalan berikut. Sila tanda ✔ pada kotak yang berkenaan.

Do you have the following condition?

Adakah anda mempunyai penyakit berikut?

Table 5 presents the questionnaire on cardio-metabolic risk factors.

**Table 5:** Cardio-metabolic risk factors BMI: body mass index Credits: Risq Atiqah Munirah binti Mohamed Mustafa

Cardio-metabolic risk factors (Faktor risiko kardio-metabolik)	Yes (Ya)	No (Tidak)
Diabetes mellitus/Kencing manis		
High blood pressure/Tekanan darah tinggi		
High cholesterol/Kolesterol tinggi		
Heart disease/Penyakit jantung		
Smoke/Merokok		
Vape/Vape		
BMI classification/Klasifikasi BMI		
Underweight/Kurang berat badan: <18.5 kg/m^2^		
Normal/Normal: 18.5-22.9 kg/m^2^		
Pre-obese (overweight)/Pre-obes (Lebih berat badan): 23-27.4 kg/m^2^		
Obese/Obes: >27.5 kg/m^2^		
Physical activity/Aktiviti fizikal		
Active/Aktif		
Inactive/Tidak aktif		
Alcohol intake/Pengambilan alkohol		
Yes/Ya - If yes, please proceed with AUDIT-C, Jika ya, sila jawab soalan AUDIT-C		
No/Tidak		

Section C: Awareness of Stroke Risk Factors and Warning Signs

Bahagian C: Tahap kesedaran mengenai faktor risiko strok dan tanda-tanda amaran strok

The questionnaire on awareness of stroke warning signs and stroke risk factors is presented in Table 6 [9].

**Table 6:** Awareness of stroke warning signs and stroke risk factors Credits: Ahmed AA, Al-Shami AM, Jamshed S, Fata Nahas AR: Development of questionnaire on awareness and action towards symptoms and risk factors of heart attack and stroke among a Malaysian population. BMC Public Health. 2019, 19:1300. 10.1186/s12889-019-7596-1 [9]. Permission was obtained to use part of the questionnaire.

Warning signs of stroke/Tanda-tanda amaran dan simptom strok			
How do you identify the warning signs and symptoms of stroke? If you think the following signs and symptoms are related to the symptoms and signs of stroke, Please answer “Yes”; if you do not think so, answer “No”; if you are unsure, answer “I do not know.” Please ✔ on the answer./Bagaimanakah anda mengenal pasti tanda-tanda amaran dan simptom strok? Jika anda fikir tanda-tanda dan symptom berikut adalah berkaitan dengan tanda-tanda dan simptom strok, sila jawab “Ya”, Jika anda tidak berfikir begitu, jawab “Tidak”; Jika anda tidak pasti, jawab “Saya tidak tahu”. Sila ✔ pada pilihan jawapan anda.	Yes/Ya	No/Tidak	I do not know/Saya tidak tahu
Do you think sudden confusion and trouble speaking or understanding speech is a stroke symptom?/Adakah anda rasa kekeliruan, masalah bercakap atau memahami pertuturan dengan tiba-tiba adalah tanda strok?			
Do you think sudden nosebleed is a stroke symptom?/Adakah anda rasa pendarahan hidung tiba-tiba adalah tanda strok?			
Do you think sudden numbness or weakness of face, arm, or leg (especially on one side of the body) is a stroke symptom?/Adakah anda rasa kekebasan atau rasa lemah tiba-tiba di muka, lengan atau kaki (terutamanya pada sebelah badan) adalah tanda strok?			
Do you think sudden trouble seeing in one or both eyes is a stroke symptom?/Adakah anda rasa mengalami kesukaran tiba-tiba untuk melihat pada satu atau kedua-dua belah mata adalah tanda strok?			
Do you think sudden vomiting is a stroke symptom?/Adakah anda rasa muntah dengan tiba-tiba adalah tanda strok?			
Do you think sudden trouble walking, dizziness, and loss of balance or coordination is a stroke symptom?/Adakah anda rasa jika tiba-tiba mengalami masalah berjalan, pening, kehilangan imbangan atau koordinasi adalah tanda strok?			
Do you think sudden severe headache with no known cause is a stroke symptom?/Adakah anda rasa sakit kepala teruk dengan tiba-tiba tanpa sebab yang diketahui adalah tanda strok?			
Do you think sudden high temperature is a stroke symptom?/Adakah anda rasa suhu yang tinggi adalah tanda strok?			
Stroke risk factors/Faktor-faktor risiko strok			
How do you identify the risk factors of stroke? Please ✔ on the answer. (Select more than one option if necessary)/Bagaimanakah anda mengenal pasti faktor-faktor risiko strok? Sila ✔ pada jawapan anda. (Anda boleh memilih lebih daripada satu)	Yes/Ya	No/Tidak	I do not know/Saya tidak tahu
Smoking/Merokok			
Cough/Batuk			
Lack of exercise/Kurang bersenam			
High blood pressure/Tekanan darah tinggi			
Heart disease/Penyakit jantung			
Family history of stroke/Sejarah keluarga strok			
High level of cholesterol/Paras kolesterol yang tinggi			
Obesity or overweight/Obesiti atau berat badan berlebihan			
Diabetes/Kencing manis			
Unhealthy diet/Diet tidak sihat			
Stress/Stres			
Excessive alcohol/Pengambilan alkohol yang berlebihan			
Atrial fibrillation (Heart beats irregular)/Jantung berdegup tidak teratur			

Section D: Health-Seeking Behaviour for Stroke (The Attitude and Beliefs about Cardiovascular Disease (ABCD) Risk Questionnaire) 

Bahagian D: Tingkah laku Terhadap Rawatan Kesihatan Strok (The Attitude and Beliefs about Cardiovascular Disease (ABCD) Risk Questionnaire - Malay version (ABCD-M))

Please ✔ on the answer, choose 1 only based on the following. 

Sila tanda ✔ pada pilihan jawapan anda. Sila pilih satu jawapan sahaja. 

Table 7 shows the questionnaire on health-seeking behavior for stroke.

**Table 7:** Health-seeking behavior for stroke (ABCD-M) ABCD-M: Attitudes and Beliefs about Cardiovascular Disease risk (Malay version) Credits: Questionnaire adapted from Mat Said Z, Tengku Ismail TA, Abdul Hamid A, Sahathevan R, Abdul Aziz Z, Musa KI: The Malay version of the attitudes and beliefs about cardiovascular disease (ABCD-M) risk questionnaire: a translation, reliability and validation study. BMC Public Health. 2022, 22:1412. 10.1186/s12889-022-13811-8 [15]. Permission was obtained to use part of the questionnaire.

Number	Item/Item	Not applicable/Tidak berkaitan	Strongly disagree/Sangat tidak bersetuju	Disagree/Tidak bersetuju	Agree/Bersetuju	Strongly agree/Sangat bersetuju
Domain 1: Perceived risk of stroke/Domain 1: Persepsi risiko strok						
1.	I feel I will suffer from a stroke sometime during my life./Saya rasa saya akan mengalami strok pada satu ketika semasa hidup saya.					
2.	It is likely that I will suffer from a stroke in the future./Ada kemungkinan saya akan mengalami strok pada masa hadapan.					
3.	It is likely that I will have a stroke sometime during my life./Ada kemungkinan saya akan mengalami strok pada satu ketika semasa hidup saya.					
4.	There is a good chance I will experience a stroke in the next 10 years./Ada kemungkinan besar saya akan mengalami strok dalam 10 tahun akan datang.					
5.	My chances of suffering from a stroke in the next 10 years are great./Kemungkinan saya mengalami strok dalam 10 tahun akan datang adalah tinggi.					
6.	It is likely that I will have a stroke because of my past and/or present behaviors./Ada kemungkinan saya akan mengalami strok kerana tingkah laku saya yang lalu dan/atau sekarang.					
7.	I am not worried that I might have a stroke./Saya tidak risau bahawa saya mungkin mengalami strok.					
8.	I am concerned about the likelihood of having a stroke in the near future./Saya bimbang mengenai kemungkinan mendapat strok tidak lama lagi.					
Domain 2: Perceived benefits/Domain 2: Persepsi kebaikan						
9.	When I exercise for at least 2 ½ hours a week, I am doing something good for the health of my heart./Apabila saya bersenam sekurang-kurangnya 2 ½ jam seminggu saya melakukan sesuatu yang baik untuk kesihatan jantung saya.					
10.	I am confident that I can maintain a healthy weight by exercising at least 2 ½ hours a week within the next two months./Saya yakin saya dapat mengekalkan berat badan yang sihat dengan bersenam sekurang-kurangnya 2 ½ jam seminggu dalam tempoh dua bulan akan datang.					
11.	When I eat at least five portions of fruit and vegetables a day, I am doing something good for the health of my heart./Apabila saya makan sekurang-kurangnya lima hidangan buah-buahan dan sayur-sayuran sehari saya melakukan sesuatu yang baik untuk kesihatan jantung saya.					
12.	Increasing my exercise to at least 2 ½ hours a week will decrease my chances of having a stroke./Meningkatkan senaman saya untuk sekurang-kurangnya 2 ½ jam seminggu dapat mengurangkan kemungkinan saya mendapat strok.					
Domain 3: Intention to change/Domain 3: Niat untuk berubah						
13.	I am thinking about exercising at least 2 ½ hours a week./Saya memikirkan untuk bersenam sekurang-kurangnya 2 ½ jam seminggu.					
14.	I intend or want to exercise at least 2 ½ hours a week./Saya berhasrat atau mahu bersenam sekurang-kurangnya 2 ½ jam seminggu.					
15.	I am not thinking about exercising for 2 ½ hours a week./Saya tidak memikirkan untuk bersenam selama 2 ½ jam seminggu.					
16.	I am confident that I can eat at least five portions of fruits and vegetables per day within the next two months./Saya yakin saya boleh makan sekurang-kurangnya lima hidangan buah-buahan dan sayur-sayuran setiap hari dalam tempoh dua bulan akan datang.					
17.	I am thinking about eating at least five portions of fruits and vegetables a day./Saya memikirkan untuk makan sekurang-kurangnya lima hidangan buah-buahan dan sayur-sayuran setiap hari.					
18.	I am not thinking about eating at least five portions of fruits and vegetables a day./Saya tidak memikirkan untuk makan sekurang-kurangnya lima hidangan buah-buahan dan sayur-sayuran setiap hari.					
Total score/Jumlah markah						
